# *PON1* hypermethylation is associated with progression of renal cell carcinoma

**DOI:** 10.1111/jcmm.14537

**Published:** 2019-08-10

**Authors:** Xin Li, Qian Yu

**Affiliations:** ^1^ Department of Pharmacy China‐Japan Union Hospital of Jilin University Changchun China

**Keywords:** 5‐Aza‐dC, DNA methylation, *PON1*, renal cell carcinoma, sunitinib, TSA

## Abstract

In this study, our aim was to exploring the influences of DNA methylation of *PON1* on cell proliferation, migration and apoptosis of renal cancer cells. The genome‐wide methylation array of renal cell carcinoma samples and adjacent tissues were obtained from the cancer genome atlas (TCGA) database. By analysing the DNA methylation and conducting the CpG islands array, methylation status expressed in renal tumour samples and normal renal tissue samples were detected. Methylation‐specific PCR (MS‐PCR) and qRT‐PCR were employed to detect the methylation level and mRNA expression of *PON1*. Wound‐healing assay, transwell assay and MTT assay were utilized to detecting the migration, invasion and proliferation abilities, respectively. The cell apoptosis was testified by Tunnel assay. In addition, the effect of *PON1* on renal cancer cells was verified by experiments in vivo. The methylation status of different genes in renal cell carcinoma samples was obtained by CpG islands arrays and hypermethylated *PON1* was selected for further study. *PON1* was down‐regulated in renal cell carcinoma tissues detected by qRT‐PCR and Western blot. Both in vitro and vivo experiments indicated that the sunitinib‐resistant in renal cancer cells could be suppressed by treat with 5‐Aza‐dC or TSA, and the effect came out more obvious after 5‐Aza‐dC and TSA co‐treatment. In detail, the demethylation of *PON1* inhibited the migration, invasion and proliferation of renal cancer cells and also arrested more cells in G0/G1 phase. The *vivo* experiment indicated that demethylated *PON1* suppressed the growth of tumour. Hypermethylated *PON1* promoted migration, invasion and proliferation of sunitinib‐resistance renal cancer cells and arrested more cells in G0/G1 phase.

## 1 INTRODUCTION

Renal cell carcinoma (RCC) was the most frequent malignancies in the kidney.^1^ Among the heterogeneous subtypes of RCC, kidney renal papillary cell carcinoma (KIRP) ranked second in the aspect of attack rate, accounting for 10%‐15%, following kidney renal clear cell carcinoma (KIRC) with incidence of 75%‐80%.^2^ Patients with RCC had a tendency to achieve a poor prognosis, and an effective prognostic indicator was also lacking for it.^3^ Because ofthese reasons, it was urgent and promising to find bio‐markers to resist this disease. In current study, we tried to revealing the internal mechanism between DNA methylation and RCC.

DNA methylation was heritable and reversible, belonged to epigenetic changes, which represent in the interface between the genome and the environment.^4^ In the procession of DNA methylation, cytosine was covalently modified by adding a methyl group to their backbone, forming a 5‐methylcytosine nucleotide (5‐mC).^5^ In addition to play an important epigenetic maker in gene silencing, DNA methylation was also involved in regulating normal growth and developmental processes such as cell differentiation, genomic imprinting and suppression of repetitive elements.^6^, ^7^, ^8^, ^9^ There have been some studies about DNA methylation and renal cancers. Malouf et al defined the epigenetic basis for proximal versus distal tubule‐derived kidney tumours.^10^ Besides, methylation‐associated genes such as *SETD2*,* KRT19* and *SFRP1* have been studied in renal cancers.^11^, ^12^, ^13^ However, the mechanism of DNA methylation in RCC remained plenty of unclear regions.

Human serum paraoxonase‐1 (PON1) is a Ca^2+^ dependent high‐density lipoprotein (HDL) associated lactonase capable of hydrolysing a wide variety of lactones, thiolactones, arylesters and cyclic carbonates.^14^ *PON1* is a kind of glycoprotein, which composed of 354 amino acids and approximate molecular mass of 43 KDa, and it retains its hydrophobic signal sequence in the N‐terminal region which enables its association with HDL.^15^ *PON1* encodes a member of the paraoxonase family of enzymes and exhibits lactonase and ester hydrolase activity. In regard to the methylation of *PON1*, hypomethylated CpGs in the promoter of *PON1* was predicted to be an underlying risk of bleeding after dual antiplatelet therapy.^16^ Moreover, recent investigations indicated that *PON1* had considerable effect on molecular disorders connected with cancer.^17^, ^18^, ^19^ Some researchers found that measurement of serum *PON1* concentration post‐radiotherapy could be an efficient prognostic biomarker and an index of the efficacy of the radiotherapy.^20^ And in this paper, we devoted to explaining the mechanism of *PON1* in KIRP.

5‐aza‐2′‐deoxycytidine (5‐Aza‐dC) was used in many DNA methylation studies. For example, Gao et al used 5‐Aza‐dC to investigating the influences of hypermethylated *MEG3* in retinoblastoma.^21^ Yan et al also used 5‐Aza‐dC, which was down‐regulated the DNA methylation of *SPARC*, to study the progression of T‐cell lymphoma.^22^ As a kind of histone deacetylase (HDAC) inhibitors, trichostatin A (TSA) could retard the growth of carcinomas of cervix, colon, rectum and other cancers in vitro.^23^ What's more, the co‐treatment of 5‐Aza‐dC and TSA showed a better effect in human gastric cancer cells.^24^ In addition, sunitinib, a multitargeted tyrosine kinase inhibitor (TKI), which currently was the standard of care for patients suffering from metastatic renal cell cancer.^25^ On the whole, we designed to exploring the impact of 5‐Aza‐dC and TSA co‐treatment in sunitinib‐resistant RCC cell.

To sum up, the aim of our study was to find the target gene affecting the progression of KIRP and to study its mechanism. Furthermore, we also testified the combination therapy in sunitinib‐resistant RCC to find a better treatment.

## 2 METHODS

### 2.1 Clinical samples

We obtained 15 pairs of RCC and corresponding para‐carcinoma tissues randomly from patients undergoing surgical treatment from May 2016 to June 2017 at China‐Japan Union Hospital of Jilin University. We got the approval from the Ethics Committee of China‐Japan Union Hospital of Jilin University to collect the samples, and we obtained the informed consent from all the patients. We took the tissues from the patients and stored them in a liquid nitrogen freezer at −80°C, preparing for subsequent experiments.

### 2.2 Cell cultures

Three human kidney cancer cell lines, including 786‐O cells, Caki‐2 cells and SKRC39 were purchased from ATCC (BeNa Culture Collection). The base medium for 786‐O cells (BeNa Culture Collection) was RPMI‐1640 Medium (GIBCO BRL). Caki‐2 cells were cultivated in MoCoy's 5a medium (GIBCO BRL) and SKRC39 were maintained in dulbecco's modified eagle medium (DMEM) (GIBCO BRL). HK‐2 cells (Cell Bank of the Chinese Academy of Sciences) were grown in a 1:1 mixture of DMEM/Ham's F 12 nutrient medium (F12). All media needed to add 10% foetal bovine serum (FBS) (GIBCO Invitrogen). Culture plates were placed at the condition of 37°C and 5% CO_2_ in an incubator.

### 2.3 Genome‐wide methylation analysis

The genome‐wide methylation array of renal cell carcinoma samples and adjacent tissues were obtained from The Cancer Genome Atlas (TCGA) database to perform the unsupervised hierarchical clustering. The ChAMP R package (http://www.bioconductor.org/packages/devel/bioc/html/ChAMP.html) was employed for methylation analysis, which contained limma‐based differential methylation protein (DMP) and Probe Lasso‐based differential methylation region (DMR) analysis function. By using the Illumina Infinium Human Methylation 450 Bead Array platform, the DNA methylation and the methylation index (MI) came out was assessed by β‐values which is calculated by β = M/[M + U] (M means methylated, U means unmethylated). And the methylation level for each CpG site were represented as β‐value ranging from 0 (U) to 1 (M).

### 2.4 Methylation‐specific polymerase chain reaction (MS‐PCR)

To perform MS‐PCR, a DNeasy Tissue Kit (Qiagen) was used to extract DNA from the tissue samples following the protocol described by the manufacturer. Then use a CpGenome DNA Modification kit (Intergene) to make the extracted DNA of 1 μg from each sample bisulfite‐conversed. In brief, dilute 1.0 μg DNA in 100 μL distilled water and use 3 M NaOH to denature it for 10 min at 45°C. Next step was to use 20 µL of 10 mmol/L hydroquinone (Sigma‐Aldrich) and 500 µL of 4.8 M sodium bisulfite (Sigma‐Aldrich) for 15 hours at 55°C to treat the DNA. We designed the MS‐PCR primers by using Methyl Primer Express software v1.0 and then amplify the PCR in a 25 μL volume containing 0.30 μL of Hot‐StarTaq Master Mix (Qiagen), 2 μg of bisulfite‐treated DNA template, and 0.5 μmol/L of each primer pair. After amplification, separate 20 μL MSP products on a 3% agarose gel containing GelRed™ Nucleic Acid Gel Stain (Biotium). The negative control was DNA‐free water blank. Table S1 showed the primers used**.**

### 2.5 Treatment of RCC cells with 5‐Aza‐dC, TSA and sunitinib

The human RCC cells was cultured in 6‐cm dishes and incubated overnight. Treat cells with 1 µmol/L 5‐Aza‐dC (Sigma‐Aldrich), 100 nmol/L TSA (Sigma‐Aldrich) and 1 μg/mL sunitinib (Pfizer). And the groups are divided into: (1), SKRC39/sunitinib cells; (2), SKRC39/sunitinib cells treated with 5‐Aza‐dC; (3), SKRC39/sunitinib cells treated with TSA; (4), SKRC39/sunitinib cells treated with 5‐Aza‐dC and TSA. Then extract DNA and mRNA or protein for QRT‐PCR and Western blot.

### 2.6 QRT‐PCR

Extract the total RNA by TRIzol reagent (Invitrogen) from tissues or cells and then use first strand synthesis kit (Thermo Fisher Scientific) to make extracted RNA reverse‐transcribe into cDNA. Carry out the qRT‐PCR by the Maxima SYBR Green qPCR Master Mix (2X) kit (Thermo Fisher Scientific) following the protocol. Specific primers were used in Table S2. The results were recorded after the cycle, and the relative gene expression was analysed by $2^{-\Delta \Delta C_{t}}$ method.

### 2.7 Western blot

The total protein was separated by RIPA lysate (Thermo Fisher Scientific). Then use BCA Kit (Sigma‐Aldrich) to quantify the protein concentration. Separate the proteins by SDS‐polyacrylamide gelelectrophoresis (SDS‐PAGE) and transfer them to a polyvinylidene fluoride (PVDF) membrane (Ruiqi). Then block the membrane with 5% (w/v) bovine serum albumin in Tris‐buffered saline (TBS) buffer at room temperature for 45 minutes and incubated first antibody anti‐*PON1* (ab24261, Abcam) and anti‐GAPDH (ab8245, Abcam) at 4°C overnight. GAPDH was used for normalization. Wash the PVDF membrane three times by TBS and incubate them with the peroxidase‐conjugated mouse anti‐goat IgG antibody (Abcam) for 4 hours at room temperature. Immunologically active proteins were visualized with an enhanced chemiluminescence system and analyse the results by using ImageJ software.

### 2.8 Wound‐healing assay

1 × 10^6^ SKRC39 cells were seeded in 6‐well plates. Then use a pipette tip to draw a straight scratch on the bottom of the plates. Wash the suspension cells twice or three times gently by PBS and take images of the scratch as baseline by the microscope. After that, the medium was replaced to the serum‐free RPMI‐1640 medium. Add different drugs into each group and take pictures of the same location again after the cells were cultured for 24 hours.

### 2.9 Transwell assay

In the transwell assay, Matrigel invasion chambers kits (Thermo Fisher Scientific) in 24‐well plates (2.5 × 10^4^ cells per well) were used following the manufacturer's instruction. Briefly, add 200 μL medium without serum containing 1 × 10^5^ cultured cells to the upper transwell chamber from each group. Add a volume of 0.5 mL of medium containing 10% FBS to the bottom chamber. After incubation at the condition of 37°C and 5% CO_2_ for 24 hours, we scraped off the cells on the upper surface of the membrane by cotton swabs. Cells that aggressed through the 8‐mm sized pores and adhered to the lower surface of the membrane were fixed with 4% paraformaldehyde and stained with 0.1% crystal violet staining solution. We needed to count at least five random microscopic fields and then take their average as the number of the cells on the bottom of the membrane.

### 2.10 MTT assay

Methylthiazolyldiphenyl tetrazolium bromide (MTT; Sigma Aldrich) was used for assessing the cell viability according to the manufacturer instructions. Place the transduced cells from the exponential phase into 96‐well plates with 0.1 mL culture medium which contained needed drugs and then incubated them for 48 hours (37°C, 5% CO_2_). After that, add MTT reagent into the plates and incubate the cells for 3 hours (37°C, 5% CO_2_). Decant the solution and add 100 μL DMSO to dissolve the purple coloured formazan crystals. Measure the absorbance of the resulting solution at 492 nm by a microplate reader (Bio Tek). Set the absorbance of the untreated culture as 100%. Calculate IC50 values using nonlinear best fit regression analysis by GraphPad Prism 6.0. And IC50 = (0.5‐b)/a. (b = constant number and a = X Coefficient). All experiments needed to be performed in triplicate and repeated for three times.

### 2.11 Flow cytometry assay

After drug treatment or transfection for 24 hours, wash the cells with pre‐cooled phosphate‐buffered saline (PBS). We used the Propidium Iodide (PI)/RNase staining kits (Thermo Fisher Scientific) to analyse cell cycle. And the apoptosis of experimental cells was detected by using annexin V‐fluorescein isothiocyanate (FITC) apoptosis detection kits (Abnova) in a FACScan instrument (BD Biosciences). What else, to better show the state of cells, we used a fluorescence microscope (IX71, Olympus) to photograph the morphological features of apoptotic cells after they were harvested and suspended in PBS containing fluorescence dye dual acridine orange/ethidium bromide (AO/EB) (AMRESCO) (Sigma‐Aldrich) and both AO and EB were at the concentration of 100 mg/L in PBS.

### 2.12 Tunnel assay

SKRC39 cells treated with four methods (sunitinib group (control group), 5‐Aza‐dC group, TSA group and 5‐Aza‐dC + TSA + sunitinib group) were stably transfected, dealt with hunger and washed twice in PBS (Thermo Fisher Scientific) at 4°C, and re‐suspended in 250 μL labelling buffer (Haoranbio). Cells from each group were stained with 5 μL annexin V/FITC and 10 μL of 20 μg/mL propidium iodine (Sigma‐Aldrich) and were incubated for 15 minutes at 37°C in the dark. The results can be observed by fluorescence microscope (ZEISS).

### 2.13 Tumour xenograft

Five‐week nude mice at the same condition were arranged as seven groups randomly, with five in each group, including normal group, sunitinib group, 5‐Aza‐dC group, TSA group, 5‐Aza‐dC + sunitinib group, TSA + sunitinib group and 5‐Aza‐dC + TSA + sunitinib group. Then inject SKRC39 cells (1 × 10^6^/200 μL PBS) subcutaneously inoculated into the nude mice at a single site. Use calipers to measure the size of tumour weekly. When the average tumour size reached about 100 mm^3^, inject the following drugs to carry the following experiments. The drugs used in this study were 5‐Aza‐dC (5 mg/kg), TSA (0.25 mg/kg) and sunitinib (2.5 mg/kg). Combinatorial treatments were performed by pre‐treatment with 5‐Aza‐dC and/or TSA for 3 d and then sunitinib was added on the 4th and 7th day. Measure the tumour volumes and tumour weights every 2 days after treatment with drugs. Fifteen days after simultaneous administration, all mice were killed and whole proteins were isolated from the xenografted tissues of the mice for Western blot analysis and immunohistochemical analysis. The Animal Care and Use Committee of China‐Japan Union Hospital of Jilin University approved all these experiments. And we obeyed the institutional ethics guidelines when we done the animal experiments.

### 2.14 Immunohistochemistry

Deparaffinize the tissue samples with xylene and then rehydrate the sections (5 mm) from tissues with ethanol. After that, to block endogenous peroxidase activity, we should immerse them in 3% hydrogen peroxide solution for 15 minutes. After rinsed with PBS, add primary antibodies against Ki67 (ab15580, Abcam) at a dilution of 1:200 and the antibody‐binding signal was detected by using the NovoLink Polymer Detection System (Leica Microsystems) and visualized with the diaminobenzidine reaction. Use haematoxylin to counterstain the sections and observe them by using a microscope.

### 2.15 Statistical analysis

We used SPSS standard version 19.0 (SPSS Inc) and GraphPad Prism 6.0 to analyse all results which came from the averages of at least three independent experiments. Statistical analyses were performed using a paired sample *t*‐test and one‐way analysis of variance (ANOVA). Data from all quantitative assays were shown as the mean ± standard and *P* < 0.05 was considered to indicate a statistically significant difference.

## 3 RESULTS

### 3.1 *PON1* presented hypermethylated and lower mRNA expressed in RCC

**Figure 1:**
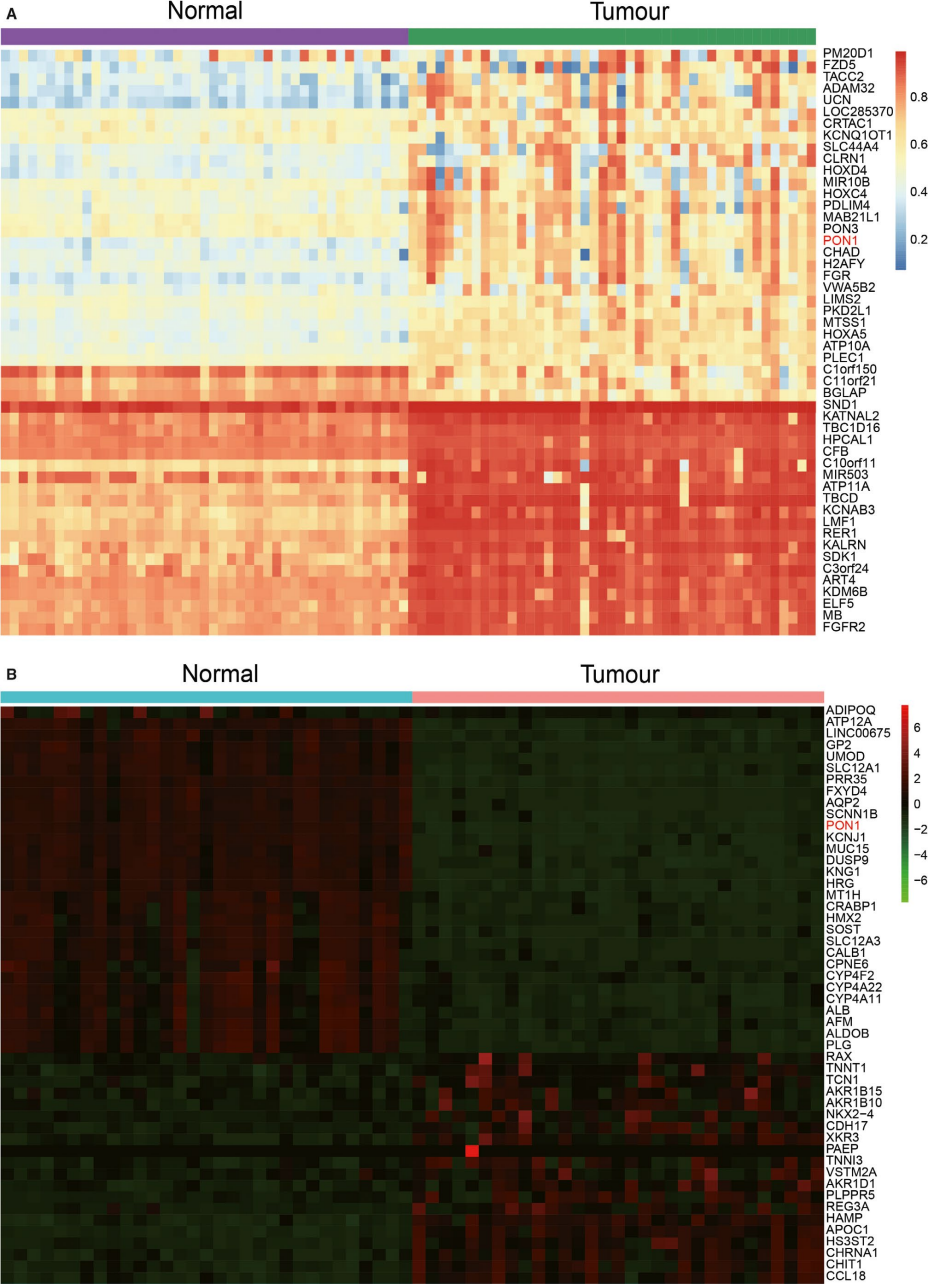
*PON1* displayed hypermethylation and lower mRNA expression level. A, The heatmap of top 50 differentially methylated genes. *PON1* showed higher methylation in tumour tissues compare with normal tissues. B, The heatmap of top 50 differentially mRNA expression level genes. *PON1* showed lower mRNA expression level in tumour tissues compared with normal tissues

**Figure 2:**
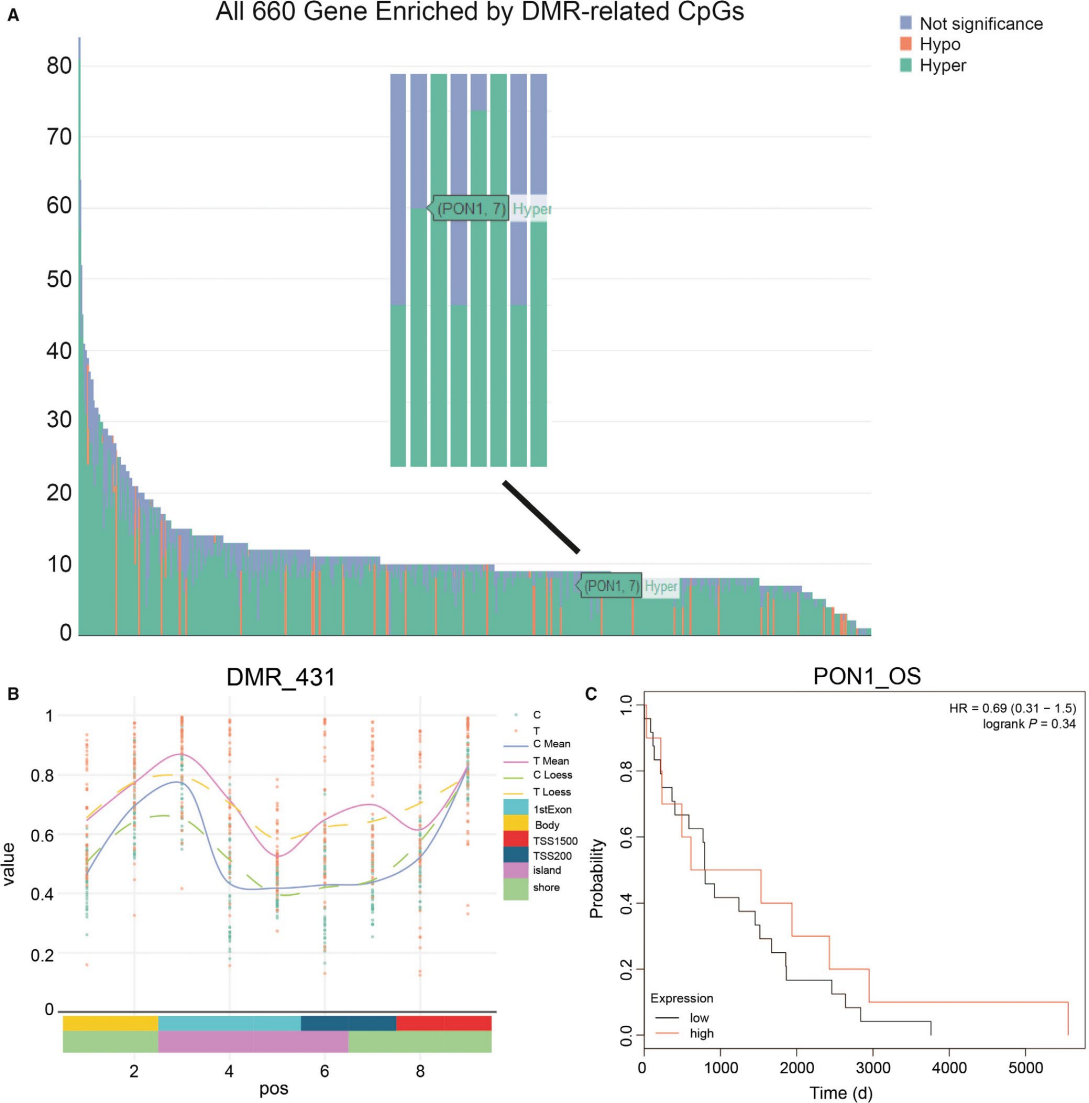
CpG analysis of *PON1* methylation status. A, All 660 Gene Enriched by DMR‐related CpGs, the *PON1* gene was hypermethylated. B, A differentially methylated region (DMR_431) of *PON1* was showed and most CpG islands on the *PON1* gene promoter were hypermethylated. C, Kaplan‐Meier analysis showed that hypermethylated *PON1* had shorter overall survival

As shown in Figure S1, the hierarchical clustering analysis screened the top 1000 differential CpG islands from 395 412 probes. In addition, Figure S2 revealed the distribution of top 1000 differentially methylated imprinted CpG sites, combining genetic and epigenetic annotation information. Methylation analysis of the paired tumour/normal tissues revealed 50 most differentially methylated genes. Compared with normal tissues, *PON1* showed obvious hypermethylation in KIRP tumour tissues (Figure 1A). The relative heatmap showed the methylation data as a standard control (from low to high methylated) in a blue‐red scale (from low to high methylation level). Analogously, differential mRNA analysis of paired tumour/normal tissues identified the top 50 differentially mRNA‐expression‐level genes in a green‐red scale (from low to high mRNA expression level) (Figure 2B). The above two heat maps revealed that *PON1* presented high methylated and low mRNA expressed in the RCC tissues.

### 3.2 Methylation status of *PON1*

The methylation status of 660 genes were detected by CpGs island analysis which revealed that *PON1* was hypermethylated among them (Figure 2A). The region of DMR_431 which was one of the differentially methylated regions of *PON1* showed that most CpG islands on the *PON1* gene were in high methylation level (Figure 2B). Among the 9 differentially methylated imprinted sites including cg01874867, cg04155289, cg05342682, cg07404485, cg17330251, cg19678392, cg24062571, cg22798737 and cg21856205, these box plots revealed that the methylation level was high in the RCC cells than in the normal cells except cg 24062571 and cg 22798737 (Figure S3). Kaplan‐Meier analysis showed that hypermethylated *PON1* had shorter lifespan generally. Calculate the *P* value by the log‐rank test (Figure 2C). Taken together, the above results showed that the high DNA methylation level of *PON1* in RCC tissues.

### 3.3 Hypermethylation status of *PON1* in RCC tissues and cells

**Figure 3:**
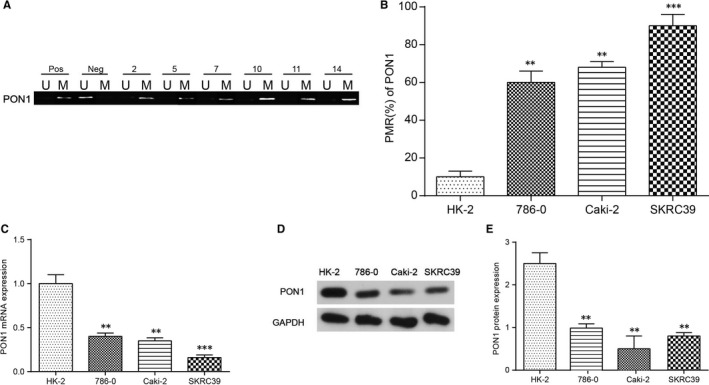
*PON1* was hypermethylated in renal tumour cells. A, *PON1* was hypermethylated in tumour tissues using methylation‐specific PCR. ‘Pos’ represented positive control; ‘Neg’ represented negative control. M: methylated; U: unmethylated. B, *PON1* was confirmed to be hypermethylated in 786‐O, Caki‐2, and SKRC39 cell lines compared with HK‐2cells. The change of DNA methylation level was maximal in SKRC39 cell line. ^**^ *P* < 0.01, ^***^ *P* < 0.001, compared with the HK‐2 cell lines. C, The mRNA levels of *PON1* in tumour cells and normal cells were analysed by real‐time PCR. ^**^ *P* < 0.01, ^***^ *P* < 0.001, compared with the HK‐2 cell lines. Each data represented mean value ± standard deviation (SD). D‐E, The protein levels of PON1 in tumour cells and normal cells were determined using Western blotting. ^**^ *P* < 0.01, compared with the HK‐2 cell lines

*PON1* in the RCC tumour tissues from 15 RCC patients was highly methylated which had the clinicopathological significance. Of the 15 RCC tumour tissues, 12 had high methylation of *PON1*, and the representative images were presented in Figure 3A. Moreover, compared with the kidney normal cell line HK‐2, *PON1* was hypermethylated in 786‐O, Caki‐2, and SKRC39 cell lines (Figure 3B). Based on RT‐PCR and Western blotting, mRNA expression and the protein level of *PON1* were analysed, respectively (Figure 3C‐E). The results indicated that the mRNA levels in the three kidney cancer cell lines (786‐O cells, Caki‐2 cells and SKRC39) were down‐regulated in comparison with HK‐2 cells and the protein level of PON1 displayed the same trend. These indicated that hypermethylation of *PON1* may result in down‐regulated the expression level of *PON1*. The trend of methylation and expression levels in SKRC39 cells was the most obvious, and SKRC39 cell line was selected for further study.

### 3.4 5‐Aza‐dC and TSA co‐treatment in SKRC39/sunitinib cells overexpressed *PON1*

**Figure 4:**
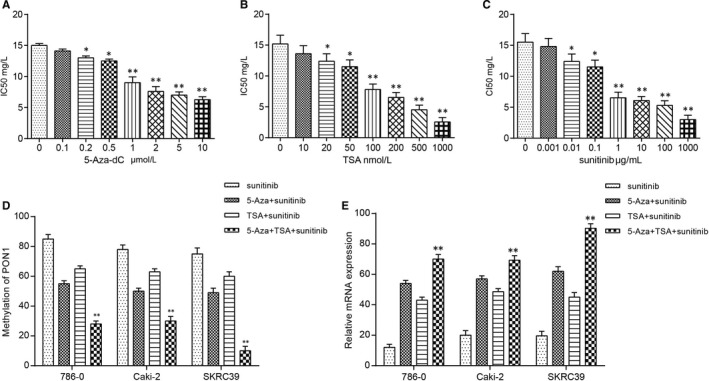
5‐Aza‐dC and TSA co‐treatment induced demethylation and re‐expression of *PON1*. A, Minimum effective dose of 5‐Aza‐dC was determined by MTT and 1 μmol/L showed difference. ^*^ *P* < 0.05, ^**^ *P* < 0.01, compared with 0 μmol/L group. B, Minimum effective dose of TSA was determined by MTT and 100 nmol/L showed difference. ^*^P < 0.05, ^**^ *P* < 0.01, compared with the 0 nmol/L group. C, Minimum effective dose of sunitinib was determined by MTT and 1 μg/mL showed difference. ^*^P < 0.05, ^**^ *P* < 0.01, compared with the 0 μg/mL group. D, 5‐Aza‐dC and TSA could decrease the methylation level of *PON1* compared with the sunitinib group and co‐treatment group had more obvious trend. ^**^ *P* < 0.01 compared with the sunitinib group. E, 5‐Aza‐dC and TSA could increase the expression level of *PON1* compared with the sunitinib group and co‐treatment group had more obvious trend. ^**^ *P* < 0.01 compared with the sunitinib group. Each data represented mean value ± standard deviation (SD)

MTT assay was employed to evaluate the different treatments on RCC and determine the concentration of reagents in the experiment. The results showed that most suitable dose of 5‐Aza‐dC and TSA were 1 μmol/L and 100 nmol/L respectively, and the concentration of 1 μg/mL sunitinib was selected for further studies (Figure 4A‐C). Based on the results of MSP and qRT‐PCR, the methylation and mRNA expression of *PON1* were verified and the results showed that the treatments of 1 μmol/L 5‐Aza‐dC and 100 nmol/L TSA in SKRC39/sunitinib cells had relative lower methylation level and higher expression level compare with the other two RCC cells (Figure 4D‐E). Collectively, these data indicated that after treated with 5‐Aza‐dC and TSA, the methylation level was decreased and expression level was increased in RCC cells. Besides that, re‐expression of *PON1* in co‐treatment 5‐Aza‐dC + TSA group showed a significant increase in SKRC39/sunitinib cells. In other words, the co‐treatment of 5‐Aza‐dC + TSA on SKRC39/sunitinib cells make *PON1* de‐methylated and re‐expressed significantly.

### 3.5 Impact of *PON1* in cell migration, invasion, proliferation and cell cycle in vitro

**Figure 5:**
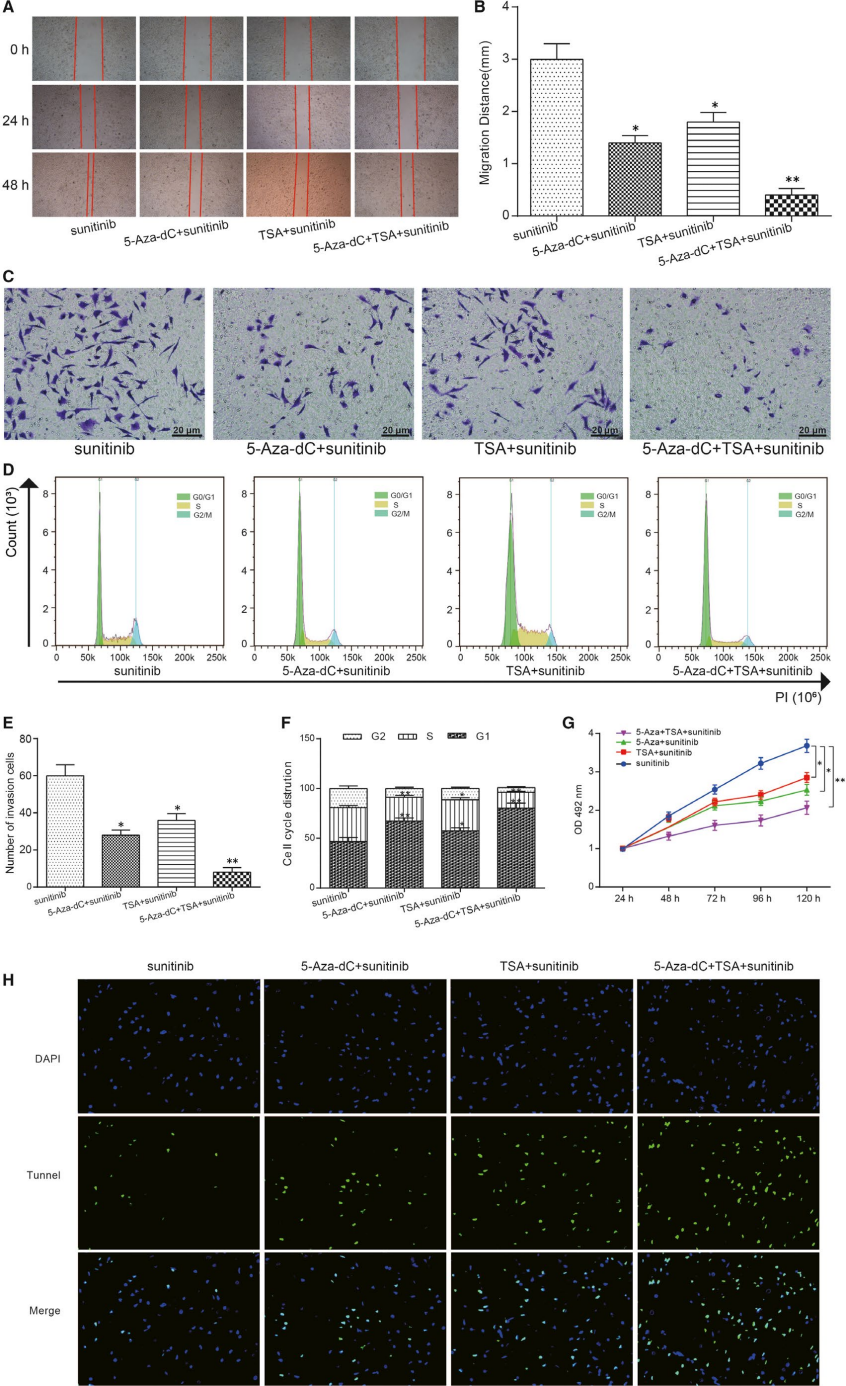
*PON1* inhibited cell migration, invasion and proliferation, and arrested more cell at the G0/G1 phase. A,B, The wound‐healing assay showed that the migration distance of 5‐Aza‐dC treatment and TSA treatment were inhibited and the co‐treatment group displayed more obvious trend. ^*^P < 0.05, ^**^ *P* < 0.01, compared with the sunitinib group. C&E, The number of invasive cells in 5‐Aza‐dC treatment and TSA treatment were decreased and co‐treatment group displayed more obvious trend. ^*^P < 0.05, ^**^ *P* < 0.01, compared with the sunitinib group. D&F, More cells were arrested at the G0/G1 phase in 5‐Aza‐dC treatment group and TSA treatment group and co‐treatment group displayed more obvious trend. ^*^P < 0.05, ^**^ *P* < 0.01, compared with the sunitinib group. G, Cell proliferation was inhibited in 5‐Aza‐dC treatment group and TSA treatment group and co‐treatment group displayed more obvious trend. ^*^P < 0.05, ^**^ *P* < 0.01, compared with the sunitinib group. H, The apoptosis cells were increased in 5‐Aza‐dC treatment group and TSA treatment group and co‐treatment group displayed more obvious trend (×40)

To exploring the role and mechanism of hypermethylated *PON1* in RCC cells, the migration, invasion, cell cycle and cell proliferation in different groups of SKRC39/sunitinib cells were analysed. The experiment groups were divided into four groups, including sunitinib group, 5‐Aza‐dC + sunitinib group, TSA + sunitinib group and 5‐Aza‐dC + TSA + sunitinib group. The cell migration abilities were detected by wound‐healing assay, and the results revealed that the migration distance in both 5‐Aza‐dC and TSA groups was inhibited better than that in the control group, but the group of 5‐Aza‐dC + TSA in SKRC39/sunitinib cells showed shorter migration distance (Figure 5A,B). Additionally, the transwell assay was performed to observing the relationship between *PON1* hypermethylation and cell aggression abilities. The results showed that the number of aggressive cells of SKRC39 cells in the group of 5‐Aza‐dC or TSA with sunitinib decreased in comparison with the control group, while the group of 5‐Aza‐dC + TSA co‐treatment in SKRC39/sunitinib cells could restrain cell aggression better (Figure 5C,E). Cell cycle was detected by flow cytometry assay, and the results revealed that treated with 5‐Aza‐dC/TSA could arrested more cells in the G0/G1 phase and co‐treatment 5‐Aza‐dC + TSA group displayed more obvious trend (Figure 5D,F). And then, cell proliferation was verified by MTT assay, proved that the cell proliferation curve of 5‐Aza‐dC + TSA in SKRC39/sunitinib cells was inhibited (Figure 5G). In addition, cell apoptosis was determined by Tunnel assay and the results confirmed that 5‐Aza‐dC and TSA could promote cell apoptosis of RCC cells (Figure 5H). These in vitro experiments above showed that re‐expression of *PON1* could restrain the migration, invasion and proliferation abilities and promote cell apoptosis in RCC cells.

### 3.6 5‐Aza‐dC + TSA + sunitinib co‐treatment group restrained RCC tumour growth in vivo

**Figure 6:**
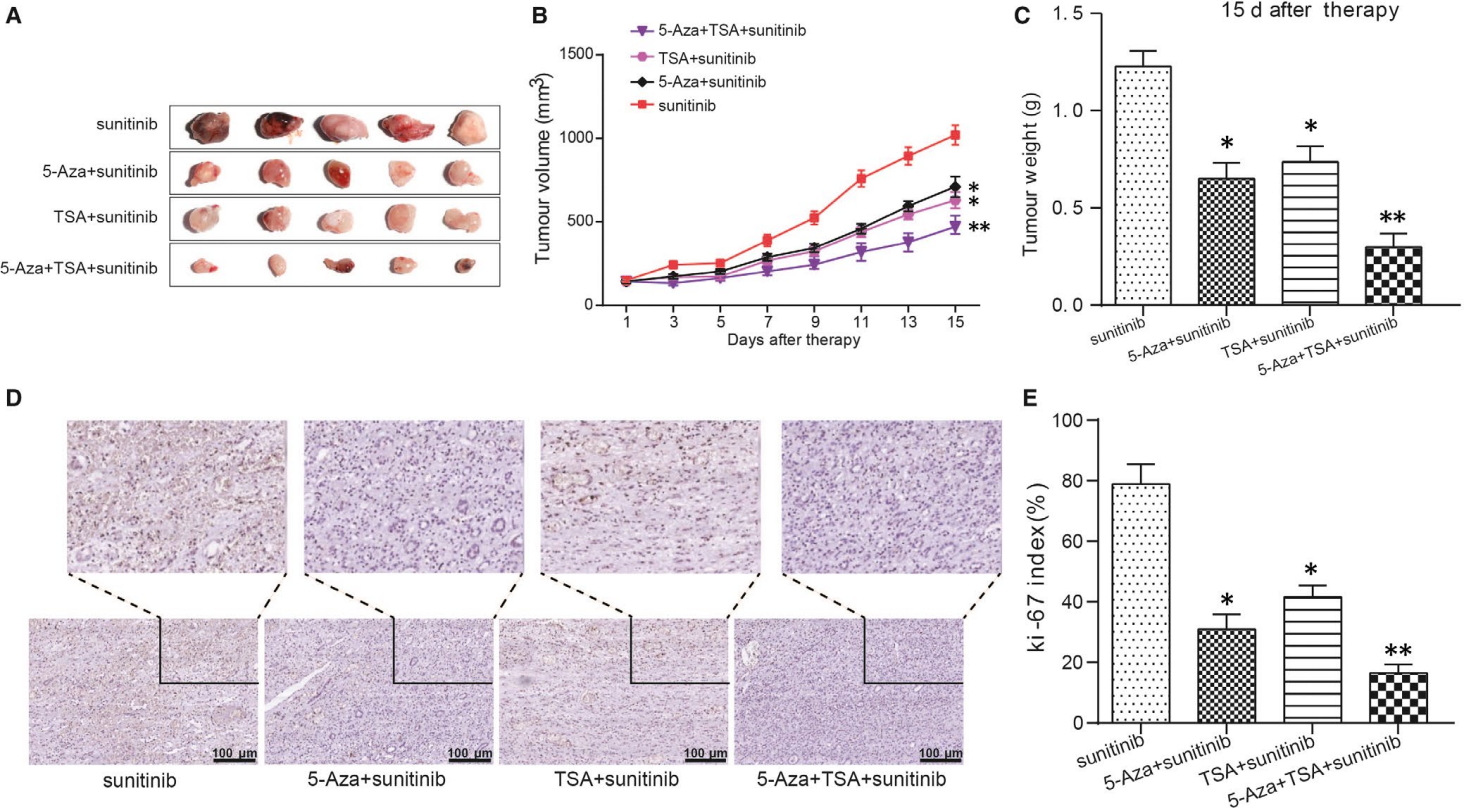
5‐Aza‐dC + TSA + sunitinib co‐treatment group suppressed tumour growth in vivo. A‐C, Tumour growth was inhibited in 5‐Aza‐dC treatment group and TSA treatment group and co‐treatment group displayed more obvious trend. ^*^P < 0.05, ^**^ *P* < 0.01, compared with the sunitinib group. D‐E, The concentration of ki67 was decreased in 5‐Aza‐dC treatment group and TSA treatment group and co‐treatment group displayed more obvious trend. ^*^P < 0.05, ^**^ *P* < 0.01, compared with the sunitinib group

To verify the curative effect of different treatment in RCC, tumour xenograft experiment and immunohistochemistry were employed for in vivo detection. The results were showed in Figure 6A‐C, and the tumour size and weight were suppressed in 5‐Aza‐dC group and TSA group. The suppression effect of co‐treatment 5‐Aza‐dC + TSA was inhibited more obvious. Immunochemical staining was conducted to verify that the index of ki67 proteins was down‐regulate significantly in the co‐treatment group of (Figure 6D‐E). These suggested that the co‐treatment group was a promising strategy for RCC therapy.

## 4 DISCUSSION

In this research, we demonstrated hypermethylated *PON1* affected on‐cogenesis of RCC. Through vivo and vitro experiments, the results showed that 5‐Aza‐dC and TSA could better block the tumour growth. The co‐treatment also made *PON1* lower methylation level and higher mRNA expression at the same time. Collectively, 5‐Aza‐dC and TSA could synergize the effect of inhibition of sunitinib‐resistant RCC tumour growth by inducing *PON1* re‐expressed.

Nowadays, DNA methylation was a research hotspot about cancer including renal cancers. The heterogeneity of DNA methylation was observed by Dugué et al, with stronger associations for risk of kidney cancer.^26^ Additionally, Kumar et al put forward the hypothesis that *IQGAP2* and *IQGAP3* were promised to be prognosis therapeutic target of specific cancers including renal cancer for the close connection of their methylation and cancers.^27^ It also reports that *RPS6KA4/MIR1237* and *AURKC* promoter regions are differentially methylated in Wilms' tumour.^28^ Analogously, we found that the methylation of *PON1* was associated with the procession of RCC which is also the base of our research.

As for*PON1* and DNA methylation, Fiorito et al found a general inverse relationship between B‐vitamins intake and DNA methylation of *PON1*.^29^ The methylation of *PON1* was also linked to vascular dementia which confirmed by Bednarska‐Makaruk et al.^30^ Interestingly, the experimental group suffered from the disease also had lower intake of vitamin B than the normal group. Although the relative studies about *PON1* and DNA methylation are not so much and it has been a forward‐looking subject due to the non‐negligible role of *PON1* played in the development of cancers including RCC.

Furthermore, the purpose of combination therapy in tumour treatment was to enhance the curative effect and reduce the occurrence of adverse reactions. According to the mechanism of anti‐tumour drugs and tumour cell proliferation kinetics, carrying out reasonable combination of drugs has been a hot area of cancer treatment in recent years. For instance, a study found that the inhibition of *CCDC69* was hypermethylated in ovarian cancer cells and might interfere with the effectiveness of combination therapy with platinum drugs, which means the combination of 5‐Aza‐dC and other anti‐cancer drugs may have better effect.^31^ To overcome drug resistance, some researchers explored a promising approach using 5‐Aza‐dC and the mTOR inhibitor everolimus in Medullary thyroid cancer cells, which showed a strong synergistic antiproliferative activity through the induction of apoptosis.^32^ Certainly, the studies of co‐treatment in renal cancer are not rare. The researchers studied how 5‐aza‐dc and paclitaxel (PTX) synergized against renal cell carcinoma (RCC).^33^ In our study, the drug combination of 5‐Aza‐dC and TSA combined with sunitinib also showed a better effect of suppressing tumorigenesis of RCC from the experimental results. Certainly, the clinical trials are needed to verify the effect.

Hypermethylation of *PON1* was present in the progression of RCC. Nonetheless, there was insufficient in the vitro experiments as we only used one RCC cell line among the three renal cell lines we researched. However, we believe this trend will be similar and further investigation will be conducted in the future. Overall, our research showed that the DNA methylation of *PON1*, which is hopeful to be a targeted biomarker in RCC, could influence the development of RCC.

## 5 CONCLUSION

In short, this is the first study on the hypermethylated *PON1* involved in the development of RCC, and it showed DNA methylation was one of the key parts in the proliferation of renal cancer cells especially the RCC cells. What's more, we have clarified the mechanism of how the DNA methylation of *PON1*, which may be a promising target for gene treatment, involved in the tumorigenesis of RCC. In addition, 5‐Aza‐dC and TSA co‐treatment for sunitinib‐resistant RCC is hopeful clinical therapy.

## CONFLICT OF INTEREST

The authors confirm that there are no conflicts of interest.

## AUTHOR CONTRIBUTIONS

XL and QY contributed to research design and the acquisition; XL analysis and interpretation of data; QY drafting the paper and revising it critically; XL and QY approval of the submission and final versions.

## ACKNOWLEDGEMENT

We'd like to express our deep appreciation to Science and Technology Development Plan Project of Jilin Provincial Science and Technology Department (No.: 20160101032JC), which offered great help in our research process.

## ETHICAL APPROVAL

The research was carried out according to the World Medical Association Declaration of Helsinki. Written informed consents were obtained from all the participants. This study approved by the Ethics Committee of China‐Japan Union Hospital of Jilin University.

## Data Availability

The data that support the findings of this study are available from the corresponding author upon reasonable request.

## References

[1] **Fabrizio** FP , **Costantini** M , **Copetti** M , et al. Keap1/Nrf2 pathway in kidney cancer: frequent methylation of KEAP1 gene promoter in clear renal cell carcinoma. Oncotarget. 2017;8:11187‐11198. 28061437 10.18632/oncotarget.14492 PMC5355256

[2] **Zhang** T , **Gong** J , **Maia** MC , **Pal** SK . Systemic therapy for non‐clear cell renal cell carcinoma. Am Soc Clin Oncol Educ Book. 2017;37:337‐342. 28561708 10.1200/EDBK_175572

[3] **Lan** H , **Zeng** J , **Chen** G , **Huang** H . Survival prediction of kidney renal papillary cell carcinoma by comprehensive LncRNA characterization. Oncotarget. 2017;8:110811‐110829. 29340018 10.18632/oncotarget.22732 PMC5762286

[4] **Ushijima** T , **Asada** K . Aberrant DNA methylation in contrast with mutations. Cancer Sci. 2010;101:300‐305. 19958364 10.1111/j.1349-7006.2009.01434.x PMC11159270

[5] **Holliday** R , **Pugh** JE . DNA modification mechanisms and gene activity during development. Science. 1975;187:226‐232. 1111098

[6] **Ikeuchi** M , **Iwase** A , **Sugimoto** K . Control of plant cell differentiation by histone modification and DNA methylation. Curr Opin Plant Biol. 2015;28:60‐67. 26454697 10.1016/j.pbi.2015.09.004

[7] **Fedoroff** NV . Presidential address. Transposable elements, epigenetics, and genome evolution. Science. 2012;338:758‐767. 23145453 10.1126/science.338.6108.758

[8] **Jones** PA , **Takai** D . The role of DNA methylation in mammalian epigenetics. Science. 2001;293:1068‐1070. 11498573 10.1126/science.1063852

[9] **Waterhouse** PM , **Wang** MB , **Lough** T . Gene silencing as an adaptive defence against viruses. Nature. 2001;411:834‐842. 11459066 10.1038/35081168

[10] **Malouf** GG , **Su** X , **Zhang** J , et al. DNA methylation signature reveals cell ontogeny of renal cell carcinomas. Clin Cancer Res. 2016;22:6236‐6246. 27256309 10.1158/1078-0432.CCR-15-1217 PMC5135666

[11] **Tiedemann** RL , **Hlady** RA , **Hanavan** PD , et al. Dynamic reprogramming of DNA methylation in SETD2‐deregulated renal cell carcinoma. Oncotarget. 2016;7:1927‐1946. 26646321 10.18632/oncotarget.6481 PMC4811507

[12] **Paiva** F , **Duarte‐Pereira** S , **Costa** VL , et al. Functional and epigenetic characterization of the KRT19 gene in renal cell neoplasms. DNA Cell Biol. 2011;30:85‐90. 20874491 10.1089/dna.2010.1108

[13] **Awakura** Y , **Nakamura** E , **Ito** N , **Kamoto** T , **Ogawa** O . Methylation‐associated silencing of SFRP1 in renal cell carcinoma. Oncol Rep. 2008;20:1257‐1263. 18949430

[14] **Rajkovic** MG , **Rumora** L , **Barisic** K . The paraoxonase 1, 2 and 3 in humans. Biochem Med (Zagreb). 2011;21:122‐130. 22135851 10.11613/bm.2011.020

[15] **Mackness** M , **Mackness** B . Human paraoxonase‐1 (PON1): Gene structure and expression, promiscuous activities and multiple physiological roles. Gene. 2015;567:12‐21. 25965560 10.1016/j.gene.2015.04.088 PMC4458450

[16] **Lei** HP , **Yu** XY , **Wu** H , et al. Effects of PON1 gene promoter DNA methylation and genetic variations on the clinical outcomes of dual antiplatelet therapy for patients undergoing percutaneous coronary intervention. Clin Pharmacokinet. 2017;57:817‐829. 10.1007/s40262-017-0595-4 28875477

[17] **Bobin‐Dubigeon** C , **Lefrancois** A , **Classe** JM , **Joalland** MP , **Bard** JM . Paired measurement of serum amyloid A (SAA) and paraoxonase 1 (PON1) as useful markers in breast cancer recurrence. Clin Biochem. 2015;48:1181‐1183. 26188919 10.1016/j.clinbiochem.2015.07.020

[18] **Ahmed** NS , **Shafik** NM , **Elraheem** OA , **Abou‐Elnoeman** SE . Association of paraoxonase‐1(Q192R and L55M) gene polymorphisms and activity with colorectal cancer and effect of surgical intervention. Asian Pac J Cancer Prev. 2015;16:803‐809. 25684529 10.7314/apjcp.2015.16.2.803

[19] **Atay** AE , **Kaplan** MA , **Evliyaoglu** O , **Ekin** N , **Isikdogan** A . The predictive role of Paraoxonase 1 (PON1) activity on survival in patients with metastatic and nonmetastatic gastric cancer. Clin Ter. 2014;165:e1‐5. 24589953 10.7471/CT.2014.1663

[20] **Arenas** M , **Garcia‐Heredia** A , **Cabre** N , et al. Effect of radiotherapy on activity and concentration of serum paraoxonase‐1 in breast cancer patients. PLoS ONE. 2017;12:e0188633. 29176871 10.1371/journal.pone.0188633 PMC5703554

[21] **Gao** Y , **Huang** P , **Zhang** J . Hypermethylation of MEG3 promoter correlates with inactivation of MEG3 and poor prognosis in patients with retinoblastoma. J Transl Med. 2017;15:268. 29287592 10.1186/s12967-017-1372-8 PMC5747938

[22] **Yan** J , **Zhang** J , **Zhang** X , et al. SPARC is down‐regulated by DNA methylation and functions as a tumor suppressor in T‐cell lymphoma. Exp Cell Res. 2018;364:125‐132. 29277504 10.1016/j.yexcr.2017.12.022

[23] **Anantharaju** PG , **Reddy** DB , **Padukudru** MA , **Chitturi** C , **Vimalambike** MG , **Madhunapantula** SV . Induction of colon and cervical cancer cell death by cinnamic acid derivatives is mediated through the inhibition of Histone Deacetylases (HDAC). PLoS ONE. 2017;12:e0186208. 29190639 10.1371/journal.pone.0186208 PMC5708809

[24] **Lin** S , **Lin** B , **Wang** X , et al. Silencing of ATP4B of ATPase H(+)/K(+) transporting beta subunit by intragenic epigenetic alteration in human gastric cancer cells. Oncol Res. 2017;25:317‐329. 28281974 10.3727/096504016X14734735156265 PMC7840950

[25] **Azar** I , **Esfandiarifard** S , **Sinai** P , **Wazir** A , **Foulke** L , **Mehdi** S . Sunitinib‐Induced Acute Interstitial Nephritis in a Thrombocytopenic Renal Cell Cancer Patient. Case Rep Oncol Med. 2017;2017:6328204. 29359059 10.1155/2017/6328204 PMC5735624

[26] **Dugue** PA , **Bassett** JK , **Joo** JE , et al. DNA methylation‐based biological aging and cancer risk and survival: Pooled analysis of seven prospective studies. Int J Cancer. 2018;142:1611‐1619. 29197076 10.1002/ijc.31189

[27] **Kumar** D , **Hassan** MK , **Pattnaik** N , **Mohapatra** N , **Dixit** M . Reduced expression of IQGAP2 and higher expression of IQGAP3 correlates with poor prognosis in cancers. PLoS ONE. 2017;12:e0186977. 29073199 10.1371/journal.pone.0186977 PMC5658114

[28] **Pereira** HS , **Soares Lima** SC , de **Faria** PS , **Cardoso** LC , **Seuanez** HN . RPS6KA4/MIR1237 and AURKC promoter regions are differentially methylated in Wilms' tumor. Front Biosci (Elite Ed). 2018;10:143‐154. 28930610 10.2741/e814

[29] **Fiorito** G , **Guarrera** S , **Valle** C , et al. B‐vitamins intake, DNA‐methylation of One Carbon Metabolism and homocysteine pathway genes and myocardial infarction risk: the EPICOR study. Nutr Metab Cardiovasc Dis. 2014;24:483‐488. 24418380 10.1016/j.numecd.2013.10.026

[30] **Bednarska‐Makaruk** M , **Graban** A , **Sobczynska‐Malefora** A , et al. Homocysteine metabolism and the associations of global DNA methylation with selected gene polymorphisms and nutritional factors in patients with dementia. Exp Gerontol. 2016;81:83‐91. 27167582 10.1016/j.exger.2016.05.002

[31] **Cui** L , **Liang** B , **Yang** Y , et al. Inhibition of coiled coil domain containing protein 69 enhances platinum‐induced apoptosis in ovarian cancer cells. Oncotarget. 2017;8:101634‐101648. 29254192 10.18632/oncotarget.21356 PMC5731902

[32] **Vitale** G , **Dicitore** A , **Pepe** D , et al. Synergistic activity of everolimus and 5‐aza‐2'‐deoxycytidine in medullary thyroid carcinoma cell lines. Mol Oncol. 2017;11:1007‐1022. 28453190 10.1002/1878-0261.12070 PMC5537710

[33] **Shang** D , **Liu** Y , **Xu** X , **Han** T , **Tian** Y . 5‐aza‐2'‐deoxycytidine enhances susceptibility of renal cell carcinoma to paclitaxel by decreasing LEF1/phospho‐beta‐catenin expression. Cancer Lett. 2011;311:230‐236. 21880414 10.1016/j.canlet.2011.08.012

