# Supplementary figures and images for: PON1 hypermethylation is associated with progression of renal cell carcinoma
Source: J Cell Mol Med. 2019 Aug 10;23(10):6646–57. doi: 10.1111/jcmm.14537 (PMC6787518; doi:10.1111/jcmm.14537)

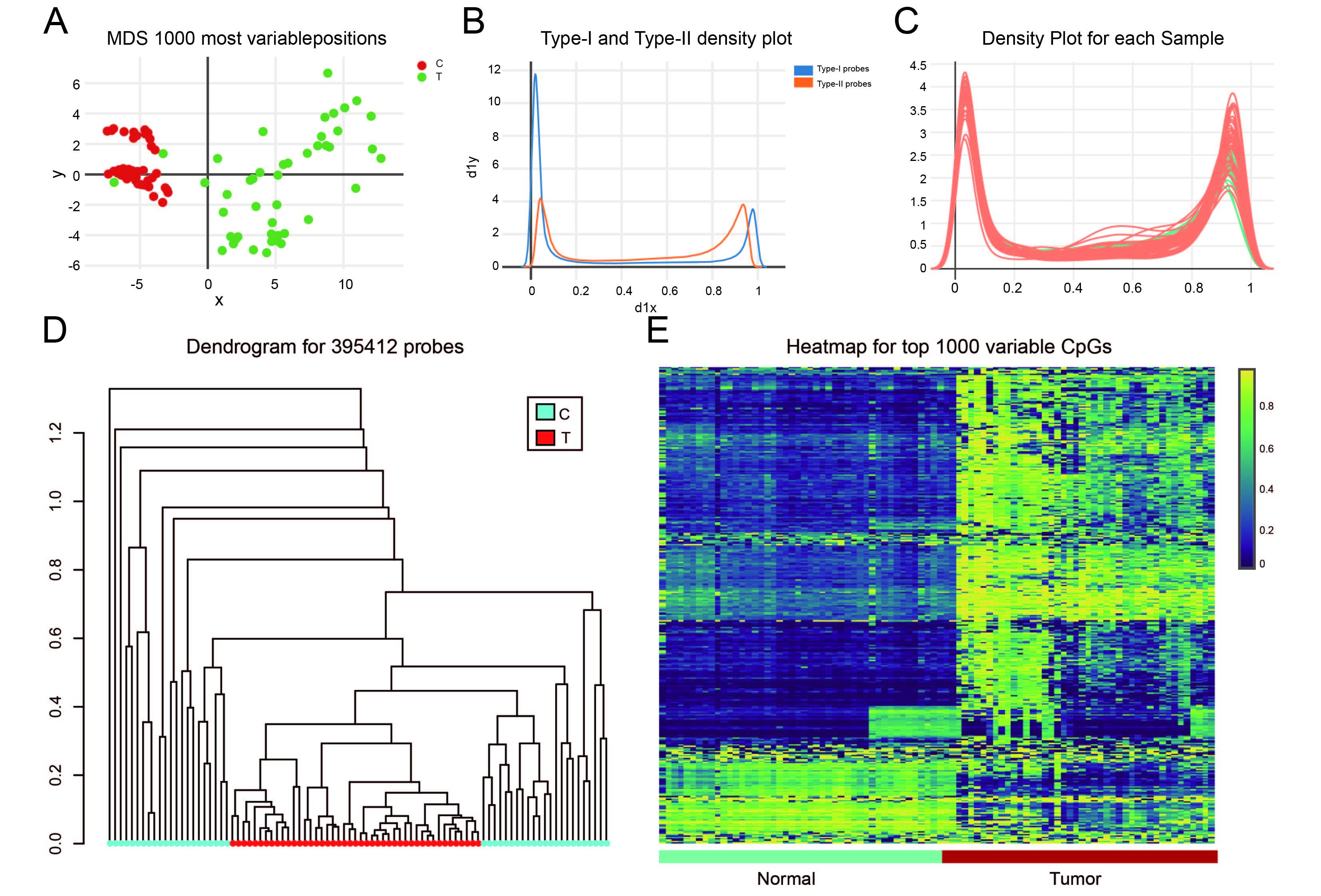

Supplement: Supplementary file 1 [file JCMM-23-6646-s001.jpg]

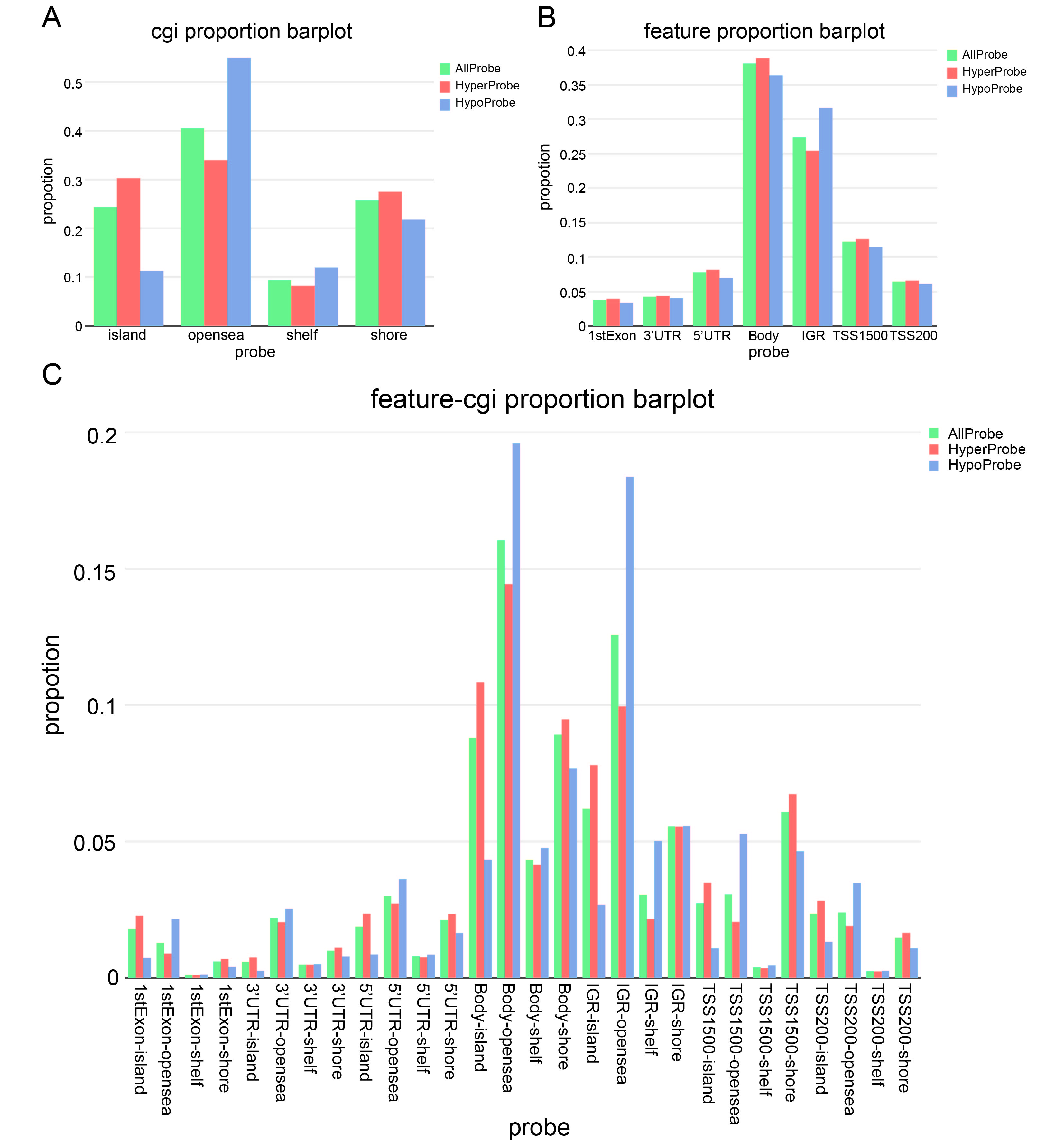

Supplement: Supplementary file 2 [file JCMM-23-6646-s002.jpg]

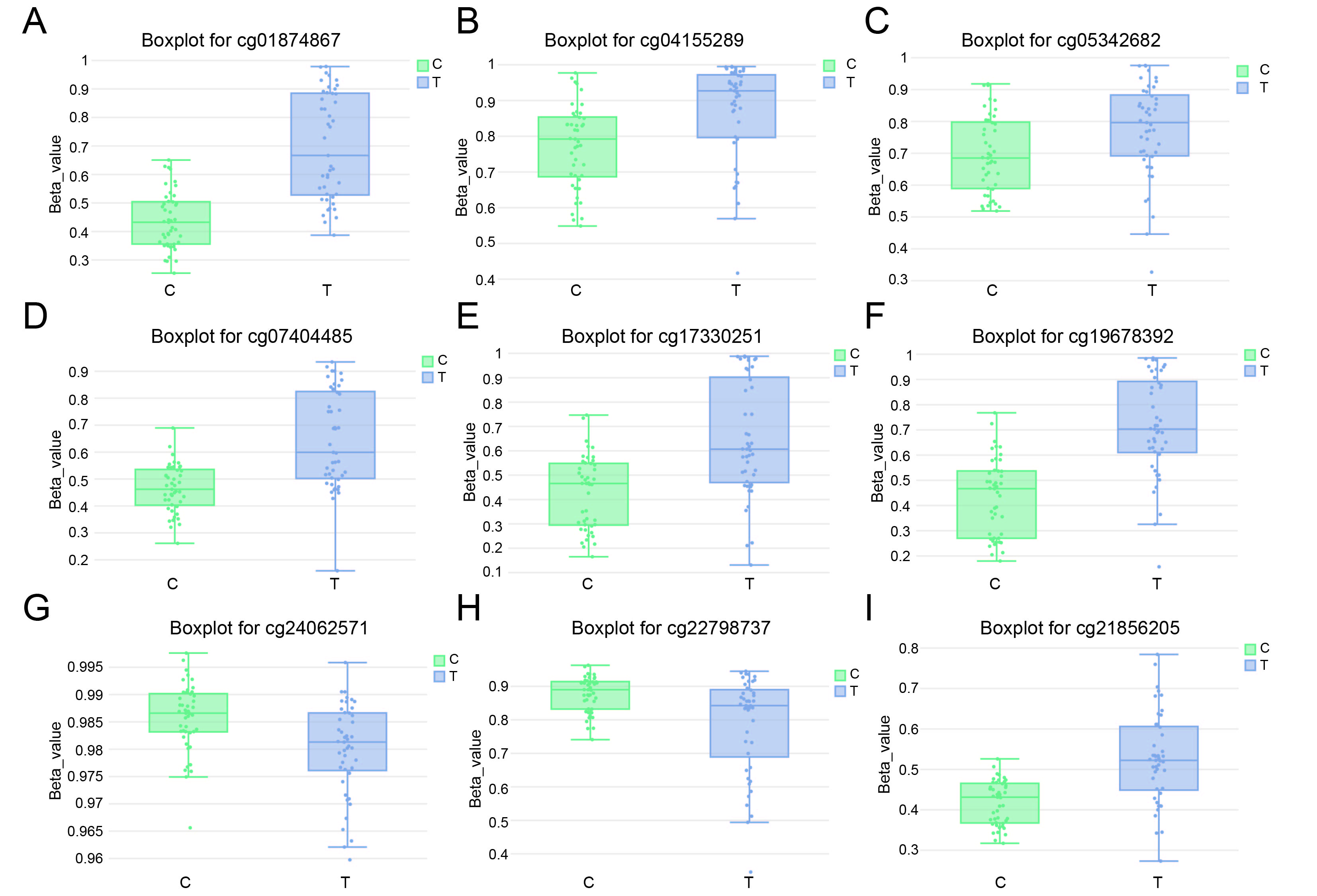

Supplement: Supplementary file 3 [file JCMM-23-6646-s003.jpg]
