# Supplementary material for: PON1 hypermethylation is associated with progression of renal cell carcinoma
Source: J Cell Mol Med. 2019 Aug 10;23(10):6646–57. doi: 10.1111/jcmm.14537 (PMC6787518; doi:10.1111/jcmm.14537)
Supplement: Supplementary file 4 [file JCMM-23-6646-s004.docx]

**Table S1** Sequences of MSP primers for qRT-PCR

| Probe | Sequence |
| --- | --- |
| Methylation |  |
| *PON1* forward | 5' TTGAGACGTAAGGATC 3' |
| *PON1* reverse | 5' AACGTAACTTACGATCA 3' |
| No Methylation |  |
| *PON1* forward | 5' TTTTGAGATGTAAGGATT 3' |
| *PON1* reverse | 5' AAAACATAACTTACAATCA 3' |
