# Supplementary material for: PON1 hypermethylation is associated with progression of renal cell carcinoma
Source: J Cell Mol Med. 2019 Aug 10;23(10):6646–57. doi: 10.1111/jcmm.14537 (PMC6787518; doi:10.1111/jcmm.14537)
Supplement: Supplementary file 5 [file JCMM-23-6646-s005.docx]

**Table S2 qRT-PCR Primer Sequences**

| Probe | Primer sequence（5’-3’） |
| --- | --- |
| *PON1* forward | 5’-TTTAATCCAGAGCTAATGAAAGCC-3’ |
| *PON1* reverse | 5’-GAAGAGTGATGTATAGCCCCAG-3’ |
| GAPDH forward | 5’-GACCTGACCTGCCGTCTA-3’ |
| GAPDH reverse | 5’-AGGAGTGGGTGTCGCTGT-3’ |
